# Dramatically Enhanced Valley‐Polarized Emission by Alloying and Electrical Tuning of Monolayer WTe_2_ *_x_* S_2(1‐_ *_x_*_)_ Alloys at Room Temperature with 1T′‐WTe_2_‐Contact

**DOI:** 10.1002/advs.202304890

**Published:** 2023-11-16

**Authors:** Wei‐Hsiang Lin, Chia‐Shuo Li, Chih‐I Wu, George R. Rossman, Harry A. Atwater, Nai‐Chang Yeh

**Affiliations:** ^1^ Department of Applied Physics California Institute of Technology Pasadena CA 91125 USA; ^2^ Graduate Institute of Photonics and Optoelectronics and Department of Electrical Engineering National Taiwan University Taipei Taiwan 106 P. R. China; ^3^ Department of Geological and Planetary Sciences California Institute of Technology Pasadena CA 91125 USA; ^4^ Department of Physics California Institute of Technology Pasadena CA 91125 USA; ^5^ Kavli Nanoscience Institute California Institute of Technology Pasadena CA 91125 USA

**Keywords:** circular dichroism, field‐effect transistors, phase transition, ternary tellurides, transition metal dichalcogenides, tunable bandgaps, valleytronics, WTe_2x_S_2(1‐x)_

## Abstract

Monolayer ternary tellurides based on alloying different transition metal dichalcogenides (TMDs) can result in new two‐dimensional (2D) materials ranging from semiconductors to metals and superconductors with tunable optical and electrical properties. Semiconducting WTe_2_ *_x_* S_2(1‐_ *_x_*_)_ monolayer possesses two inequivalent valleys in the Brillouin zone, each valley coupling selectively with circularly polarized light (CPL). The degree of valley polarization (DVP) under the excitation of CPL represents the purity of valley polarized photoluminescence (PL), a critical parameter for opto‐valleytronic applications. Here, new strategies to efficiently tailor the valley‐polarized PL from semiconducting monolayer WTe_2_ *_x_* S_2(1‐_ *_x_*_)_ at room temperature (RT) through alloying and back‐gating are presented. The DVP at RT is found to increase drastically from < 5% in WS_2_ to 40% in WTe_0.12_S_1.88_ by Te‐alloying to enhance the spin‐orbit coupling. Further enhancement and control of the DVP from 40% up to 75% is demonstrated by electrostatically doping the monolayer WTe_0.12_S_1.88_ via metallic 1T′‐WTe_2_ electrodes, where the use of 1T′‐WTe_2_ substantially lowers the Schottky barrier height (SBH) and weakens the Fermi‐level pinning of the electrical contacts. The demonstration of drastically enhanced DVP and electrical tunability in the valley‐polarized emission from 1T′‐WTe_2_/WTe_0.12_S_1.88_ heterostructures paves new pathways towards harnessing valley excitons in ultrathin valleytronic devices for RT applications.

## Graphical abstract

The degree of valley polarization at room temperature is found to increase drastically from <5% in monolayer WS_2_ to 40% in monolayer WTe_2_ *_x_* S_2(1‐_ *_x_*_)_ due to enhanced spin‐orbit coupling, and is further enhanced to 75% by electrostatically gating the 1T′‐WTe_2_/WTe_0.12_S_1.88_ heterostructures, where the use of 1T′‐WTe_2_ substantially lowers the Schottky barrier height and weakens the Fermi‐level pinning.

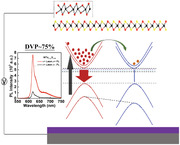

## 1 Introduction

Monolayer 1H‐phase transition metal dichalcogenides (TMDs) such as WS_2_ are direct band gap semiconductors that consist of an atomic layer of tungsten sandwiched between a top and a bottom layer of sulfur atoms that are arranged in their respective hexagonal lattice structure.^[^ ^1^, ^2^ ^]^ The band structures of monolayer TMDs^[^ ^3^, ^4^, ^5^, ^6^, ^7^ ^]^ consist of two inequivalent K (−K) valleys in the hexagonal Brillouin zone. The strong spin‐orbit coupling and broken inversion symmetry in monolayer TMDs result in a large energy splitting between the top spin‐up (spin‐down) valence band and the bottom spin‐down (spin‐up) valence band in the K (−K) valley via the preservation of time reversal symmetry.^[^ ^8^, ^9^, ^10^, ^11^, ^12^, ^13^ ^]^ Given that both of the Berry curvature and orbital magnetic moment are odd under the time‐reversal symmetry operation, one can selectively populate excitons in different valleys (K or −K) by means of circularly polarized light (CPL), where CPL with positive helicity (σ^+^) couples to the K valley and that of the negative helicity (σ^−^) couples to the −K valley according to valley‐dependent optical selection rules.^[^ ^14^, ^15^, ^16^ ^]^ However, the degree of valley polarized emission in monolayer TMDs under CPL depends strongly on the intervalley scatterring time and exciton lifetimes. Therefore, understanding the processes that govern the exciton lifetimes and the associated degree of valley polarization is essential for assessing the emergent applications of valley‐polarized excitons in devices.^[^ ^17^ ^]^ Various strategies aiming at enhancing the valley polarization by further breaking the spatial‐inversion symmetry have been proposed, including applying magnetic fields, chemical doping of magnetic elements, and employing magnetic proximity effects.^[^ ^17^, ^18^, ^19^, ^20^, ^21^, ^22^ ^]^ However, with respect to these methods for enhancing the valley polarization, the efficiency of applying an external magnetic field is extremely low for valley polarization (≈0.3 meV per Tesla); magnetic doping suffers from the formation of inhomogeneously distributed dopant clusters; and magnetic proximity effects are easily diminished by the valley submergence. Alternative approaches by electrical and optical control of the valley polarization in TMDs at room temperature and under off‐resonance conditions would be more practical and desirable.^[^ ^13^, ^17^ ^]^ Towards this goal, carrier doping, including chemical and physical approaches, appears to be an efficient way to manipulate the valley polarization, because excess carriers introduced in the TMDs not only tailor the exciton species but also modify the valley polarization dynamics considerably. Chemical doping is known to be an effective and convenient method to modify the carrier concentrations and electronic bandstructures in monolayer TMD materials, which can induce shifts in the Fermi level as well as modifications to the electronic, optical, and valley polarization properties. Physcial doping, via either electrostatic carrier doping by gating or light excitation by creating electron‐hole pairs, can also induce valley polarization enhancement through stronger screening of the Coulomb interaction by excess carriers, which helps suppress the intervalley scattering.^[^ ^17^ ^]^

For effective manipulation of the valley degrees of freedom in semiconducting monolayer TMDs by electrostatic doping, it is essential to address the interfacial issues of Fermi level pinning and Schottky barrier heights when making electrical contacts to the TMDs. To date, several approaches to circumvent these issues in TMD‐based field effect transistors (FETs) have been reported, including the use of a low work function (WF) metal for the electrical contact,^[^ ^23^, ^24^ ^]^ the use of a Fermi‐level de‐pinning layer,^[^ ^25^, ^26^ ^]^ and various techniques of molecule/chemical doping of TMDs.^[^ ^27^, ^28^, ^29^, ^30^, ^31^, ^32^ ^]^ Developing heterostructures that consist of a two‐dimensional (2D) van der Waals (vdW) metal as the top contact material on a 2D semiconductor is another approach to lower the Schottky barrier height (SBH).^[^ ^33^, ^34^, ^35^, ^36^ ^]^ For this purpose, a natural material for consideration is graphene.^[^ ^37^ ^]^ However, deposition of another metallic layer on graphene is required for electrical characterizations, and the carrier injection efficiency generally varies, depending on the metal deposited. Alternatively, the metallic 1T′‐phase WTe_2_ with a low WF^[^ ^38^ ^]^ and a vdW clean surface^[^ ^39^ ^]^ may be considered as an efficient electron‐type (*n*‐type) contact material for 2D semiconductors. However, there have not been extensive studies to date on using the 1T′‐phase WTe_2_ as the metal contact to lower the contact resistance of TMD‐based devices due to the challenges of material preparation and material stability.^[^ ^40^, ^41^, ^42^, ^43^ ^]^

**Figure 1:**
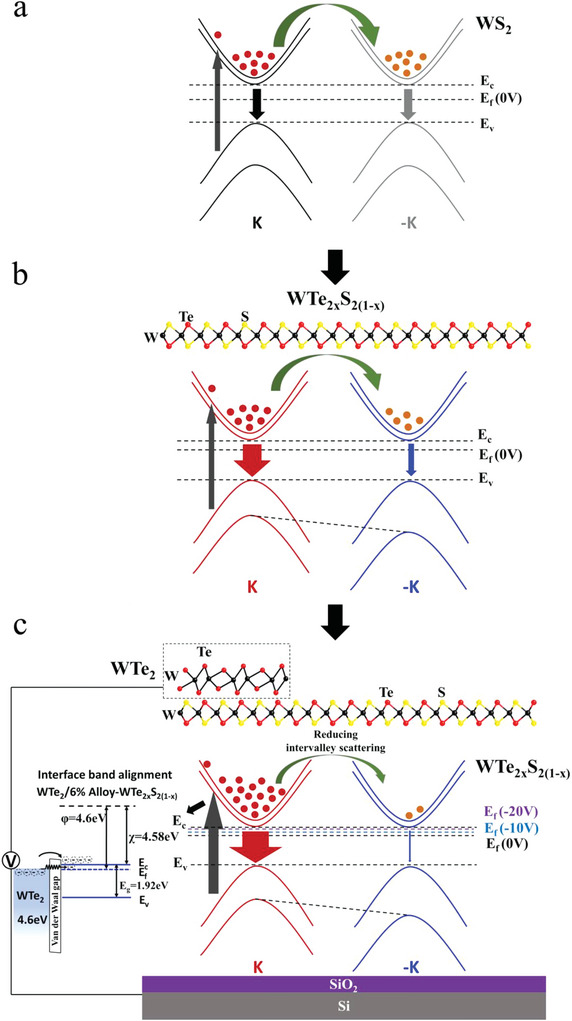
Proposed mechanism for tailoring the valley polarized PL of gated WTe_2_/6%‐WTe_2_ *_x_* S_2(1‐_ *_x_*_)_ heterostructure: a) Schematics of the energy bands of monolayer WS_2_ under right‐handed CPL with significant intervalley scattering and therefore comparable decay rates for both *σ* ^+^ and *σ* ^−^ excitons. b) Schematics of the energy bands of monolayer 6%‐WTe_2_ *_x_* S_2(1‐_ *_x_*_)_ under right‐handed CPL, showing a significantly increased decay rate for *σ* ^+^ excitons. c) Schematics of the energy bands of a gated 6%‐WTe_2_ *_x_* S_2(1‐_ *_x_*_)_/WTe_2_ heterostructure, showing both an increased decay rate of *σ* ^+^ excitons and suppressed intervalley scattering due to carrier doping‐induced screening of the long‐range electron‐hole interaction.

In this study, ternary WTe_2_ *_x_* S_2(1‐_ *_x_*_)_ (0 ≤ *x* ≤ 1) alloys were synthesized via chemical vapor deposition in a one‐step synthesis process to produce high‐quality 2D semiconductors of tunable bandgaps for high‐performance eletronic devices. By alloying Te into tungsten disulfide WS_2_, the WF of the ternary WTe_2_ *_x_* S_2(1‐_ *_x_*_)_ (0 ≤ *x* ≤ 1) alloy could be tuned to match that of the 2D contacts as the source (S) / drain (D) electrodes in the FET structure to reduce the SBH. These monolayer ternary WTe_2_ *_x_* S_2(1‐_ *_x_*_)_ (0 ≤ *x* ≤ 1) alloys evolved from the semiconducting 1H phase to the metallic 1T′ phase, depending on the Te concentration (*x*). X‐ray photoelectron spectroscopic (XPS) characterizations confirmed the existence of W, S, and Te with controlled ratios. The optical bandgap of the WTe_2_ *_x_* S_2(1‐_ *_x_*_)_ alloy could be tuned from 2 to 1.65 eV in the 1H semiconducting phase and then dropped down to 0 in the 1T′ metallic phase. The FET devices based on monolayer WTe_2_ *_x_* S_2(1‐_ *_x_*_)_ alloys revealed characteristics that confirmed the 1H phase being n‐type semiconductors and the 1T′ phase being a metal. Moreover, the use of WTe_2_ metallic contacts with a WF close to the band edge of the WTe_0.12_S_1.88_ alloy resulted in WTe_2_ *_x_* S_2(1‐_ *_x_*_)_‐based FETs with excellent electronic characteristics, including a high electron carrier mobility up to 50 cm^2^V^−1^S^−1^ and an on/off current ratio up to 10^6^. Furthermore, it has been reported that valley polarization can be tuned by doping,^[^ ^44^ ^]^ defects,^[^ ^13^, ^45^ ^]^ and alloying engineering.^[^ ^46^ ^]^ In particular, alloying with heavier elements can modify the valley polarization by enhancing the spin‐orbit coupling (SOC). Therefore, it is worth investigating how the valley polarized emission from WTe_2_ *_x_* S_2(1‐_ *_x_*_)_ alloys under CPL evolves with the concentration of Te. We note that the degree of valley polarization (DVP) for as‐grown monolayer WS_2_ is typically very low (<5%) at RT due to significant phonon‐ and defect‐induced inter‐valley scattering (**Figure** **1**a), where the DVP value (*P*_DVP_) is defined by the following expression:

(1) $$P_{\mathrm{DVP}}=\frac{I\left(\sigma^{+}\right)-I\left(\sigma^{-}\right)}{I\left(\sigma^{+}\right)+I\left(\sigma^{-}\right)}$$

with *I* (*σ* ^+^) and *I* (*σ* ^−^) denoting the right‐handed (RH) and left‐handed (LH) circular polarization‐resolved photoluminescence (PL) intensity, respectively. In contrast, the *P*_DVP_ values in monolayer ternary alloys WTe_2_ *_x_* S_2(1‐_ *_x_*_)_ with *x* > 0 were found to be tunable and were enhanced up to 40% under the excitation of right‐handed circularly polarized (RCP) light. The underlying mechanism for tailoring the valley‐polarized PL of monolayer 1H‐ternary WTe_2_ *_x_* S_2(1‐_ *_x_*_)_ alloys may be attributed to the enhanced SOC strength and broken mirror symmetry by mixing the Te‐S species, as schematically shown in Figure 1b. The stronger SOC of the Te atoms than that of the S atoms can increase the spin‐orbit energy splitting (Δ_SO_) so that Δ_SO_ (WTe_2_) = 484 meV and Δ_SO_ (WS_2_) = 412 meV.^[^ ^42^ ^]^ Additionally, by applying a back‐gated voltage *V*_G_ to WTe_2_ *_x_* S_2(1‐_ *_x_*_)_‐based FETs with 1T′‐WTe_2_ as the contact electrodes, the resulting DVP values were found to be further enhanced from 40% for *V*_G_ = 0 up to ≈75% for *V*_G_ = −20 V. This finding suggests that modulating the carrier doping level can enhance the valley polarization by screening the long‐range electron‐hole exchange interactions, thus reducing the momentum‐dependent intervalley scattering, as shown in Figure 1c. Overall, we have demonstrated successfully tuning and drastically enhancing the DVP values in semiconducting monolayer 1H‐TMDs at RT by combined strategies of chemically alloying and electrically gating the monolayer TMD‐based FETs with electrodes of reduced SBH and weakened Fermi level pinning. Our approach thus offers new opportunities toward developing realistic valley‐dependent optoelectronic devices for energy‐efficient information processing at room temperature.

## 2 Results and Discussion

**Figure 2:**
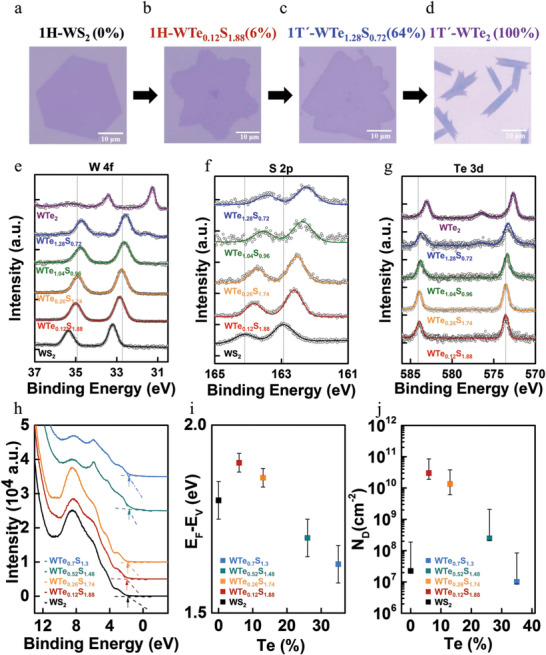
Optical microscopic images, chemical compositions and carrier doping of monolayer WTe_2_ *_x_* S_2(1‐_ *_x_*_)_ alloys for a) 1H‐WS_2_, b) 1H‐WTe_0.12_S_1.88_, c) 1T′‐ WTe_1.28_S_0.72_, d) 1T′‐ WTe_2_. XPS spectra of monolayer WTe_2_ *_x_* S_2(1‐_ *_x_*_)_ alloys with *x* = 0, 0.06, 0.13, 0.52, 0.64, and 1, respectively: e) XPS W 4f spectra, f) XPS S 2p spectra, and g) XPS Te 3d spectra. h) Valence band spectra with increasing Te doping concentration in WTe_2_ *_x_* S_2(1‐_ *_x_*_)_ alloys. i) (*E*_F_ − *E*_V_) values extracted from the UPS spectra in h) for WTe_2_ *_x_* S_2(1‐_ *_x_*_)_ alloys with different *x*‐values. j) Electron doping concentration versus Te doping concentration in WTe_2_ *_x_* S_2(1‐_ *_x_*_)_ alloys by using the (*E*_C_ − *E*_F_) values extracted from ultraviolet photoelectron spectroscopy (UPS) and PL spectra as explained in the main text.

The experimental setup for the growth of monolayer WTe_2_ *_x_* S_2(1‐_ *_x_*_)_ (0 ≤ *x* ≤ 1) alloys is schematically depicted in Supporting Information Figure S1a (see details of the synthesis process in the Experimental Section). By tuning the ratios of the chalcogen precursors and that of the Ar/H_2_ gas flow, we were able to synthesize both 1H and 1T′ phases of monolayer WTe_2_ *_x_* S_2(1‐_ *_x_*_)_ (0 ≤ *x* ≤ 1). **Figure** **2**a–d show typical optical microscopy (OM) images of the 1H and 1T′ WTe_2_ *_x_* S_2(1‐_ *_x_*_)_ monolayers. When the chalcogen ratio (Te/S) increased from 1 to 7 and the Ar/H_2_ ratio increased from 80/40 to 80/50, monolayer 1T′ WTe_2_ *_x_* S_2(1‐_ *_x_*_)_ was obtained. The reactivity of the Te with WO_3_ was far lower than that of S with WO_3_, so the usage of a large amount of Te precursors and higher H_2_ gas flow was necessary to ensure that Te could be incorporated into the WTe_2_ *_x_* S_2(1‐_ *_x_*_)_ matrix to form the 1T′ phase.

**Figure 3:**
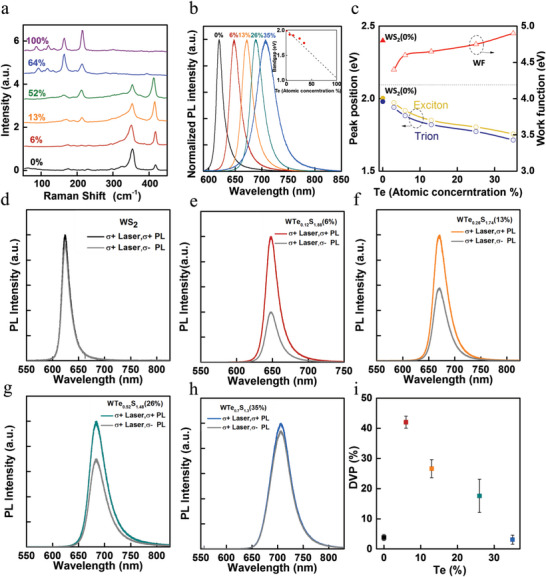
Raman, PL and valley‐polarized PL spectra of monolayer WTe_2_ *_x_* S_2(1‐_ *_x_*_)_ alloys: a) Raman spectra of the alloys with *x* = 0, 0.06, 0.13, 0.52, 0.64 and 1. b) PL spectra of monolayer WTe_2_ *_x_* S_2(1‐_ *_x_*_)_ alloys with *x* = 0, 0.03, 0.06, 0.13, 0.26, and 0.35, respectively. The inset shows the corresponding composition‐dependent (*x*) bandgap of the WTe_2_ *_x_* S_2(1‐_ *_x_*_)_ alloys, as determined by PL. c) The peak positions of A‐excitons and trions, and the corresponding work function of monolayer WTe_2_ *_x_* S_2(1‐_ *_x_*_)_ alloys with *x* = 0, 0.06, 0.13, 0.26, and 0.35, respectively. Circularly polarized PL spectra of monolayer WTe_2_ *_x_* S_2(1‐_ *_x_*_)_ alloys under the excitation of *σ* ^+^ light (514 nm) at RT: d) WS_2_, e) WTe_0.12_S_1.88_, f) WTe_0.26_S_1.74_, g) WTe_0.52_S_1.48_, and h) WTe_0.7_S_1.3_. (i) DVP versus increasing Te doping concentration on WTe_2_ *_x_* S_2(1‐_ *_x_*_)_ alloys extracted from (d) to (h), showing much enhanced DVP in WTe_0.12_S_1.88_, WTe_0.26_S_1.74_ and WTe_0.52_S_1.48_ relative to that in WS_2_.

X‐ray photoelectron spectroscopic (XPS) was used to investigate the chemical composition of the WTe_2_ *_x_* S_2(1‐_ *_x_*_)_ (0 ≤ *x* ≤ 1) alloys synthesized by atmosphere‐pressure chemical vapor deposition (APCVD) and to evaluate the electron doping concentration as a function of the Te doping concentration. The core level spectra were calibrated via fitting adventitious carbon at 284.8 eV. The high‐resolution spectra of W 4f, S 2p, and Te 3d peaks are shown in Figure 2e–g. For 1H‐phase WS_2_, the corresponding binding energies of the W 4f *_7/2_* and W 4f *_5/2_* peaks were located at 33.2 and 35.3 eV, respectively, and the binding energies for the S 2p*_3/2_* and S 2p*_1/2_* peaks were located at 162.9 and 164.2 eV, respectively, which were all consistent with the values reported previously.^[^ ^47^ ^]^ By tuning the mass ratios of Te and S powder from 1 to 100 with specific H_2_ flow rates from 40 to 60 sccm during the synthesis process, W‐Te bonds at 573.8 eV (Te 3d_5/2_) and 584.1 eV (Te 3d_3/2_) appeared in the spectra, which provided direct evidence for Te doping into the original WS_2_ crystal lattice. The binding energy of W 4f and S 2p peaks displayed a downshift ≈0.4 eV in the alloy with *x* = 13%, indicating that Te doping resulted in reduced electronegativity. When a structural phase transition occurred at a higher stoichiometric ratio (*x* > 0.5), the binding energies of W 4f, S 2p, and Te 3d all shifted to lower energy states concurrently. For 1T′‐phase WTe_2_, the main W 4f peaks at 31.28 eV (4f_7/2_) and 33.44 eV(4f_5/2_) and the Te 3d peaks located at 572.6 (3d_5/2_) and 583 eV (3d_3/2_) were assigned to the W–Te bond. The chemical stoichiometry information mentioned above directly indicated that the mole fraction of Te and the structural evolution between the 1H and 1T′ phases could be tuned by changing the mass ratio of Te and S powder together with specific H_2_ concentrations during the APCVD growth. Furthermore, the distinct binding energy redshifts of W 4f, Te 3d, and S 2p with increasing Te concentration up to *x* = 35% indicate that the Fermi level moved downward closer to the valence band and hence the p‐type doping of Te into the WS_2_ lattice, which was further corroborated by measurements of the increasing work function of WTe_2_ *_x_* S_2(1‐_ *_x_*_)_ with *x* for 0 < *x* ≤ 0.35 by ultraviolet photoelectron spectroscopy (UPS, to be further elaborated below), as shown in the inset of **Figure** **3**c. Both of the XPS and UPS showed that Te as the p‐type dopant doped in the n‐type semiconductor.

To quantitatively evaluate the changes in the electron carrier concentration with Te doping, which plays a critical role in determining the DVP of 1H‐ternary WTe_2_ *_x_* S_2(1‐_ *_x_*_)_ alloys (0 < *x* ≤ 0.35), we need to evaluate the (*E*_c_ − *E*_F_) values of WTe_2_ *_x_* S_2(1‐_ *_x_*_)_, where *E*_c_ and *E*_F_ denote the conduction band edge energy and the Fermi level, respectively. We employed the UPS studies to extract the (*E*_F_ − *E*_v_) values and the PL measurements to obtain the optical bandgap *E*_emission_ values for WTe_2_ *_x_* S_2(1‐_ *_x_*_)_ (0 < *x* ≤ 0.35), where *E*_v_ denotes the valance band edge energy, and the bandgap between the conduction and valence band edges is given by *E*_g_ ≡ (*E*_c_ − *E*_v_) = *E*_emission_ + *E*_binding_, where *E*_binding_ represents the binding energy of A‐excitons. As shown in Figure 2h, linear extraction of the valence band edge tail was used to determine the (*E*_F_ − *E*_v_) values. We found that the (*E*_F_ − *E*_v_) value first increased from 1.8 eV for *x* = 0 to 1.9 eV for *x* = 6%, and then steadily decreased with increasing *x* down to 1.57 eV for *x* = 35%, as shown in Figure 2h,i.

Next, using the optical bandgap *E*_emission_ of the 1H‐WTe_2_ *_x_* S_2(1‐_ *_x_*_)_ obtained from the PL measurements (Figure 3b), and noting that *E*_emission_ = (*E*_c_ − *E*_v_) − *E*_binding_ = (*E*_c_ − *E*_F_) + (*E*_F_ − *E*_v_) − *E*_binding_ where (*E*_F_ − *E*_v_) were given by the UPS studies as mentioned above, we derived the (*E*_c_ − *E*_F_) values for the 1H‐WTe_2_ *_x_* S_2(1‐_ *_x_*_)_ by using *E*_binding_ = Ry (*µ*/*m*_e_) (*ε*_0_/*ε*_s_)^2^ ∼ 0.1 eV, with Ry being the Rydberg energy 13.6 eV, *µ* ∼ 0.178 *m*_e_ the reduced mass of A‐excitons, *m*_e_ the free electron mass, and (*ε*_s_/*ε*_0_) ∼ 5 the dielectric constant of the substrate. After the (*E*_c_ − *E*_F_) values were determined as a function of the Te doping, the electron doping concerntrations (*N*_D_) may be calculated by using the effective density of state (*N*_C_) near the bottom of the conduction band for two‐dimensional electrons

(2) $$N_{C}=\left(4\pi m_{e}^{*}/h^{2}\right)$$

where *h* is the Planck constant. Thus, we obtained *N*_D_ from the following expression

(3) $$N_{D}=N_{C}k_{B}T\ln\left[1+\exp\left(-\frac{E_{c}-E_{F}}{k_{B}T}\right)\right]$$

where *T* is the temperature and *k*_B_ is the Boltzmann constant. The results of the *N*_D_ analysis are presented in Figure 2j, showing that *N*_D_ decreases from (3 × 10^10^) cm^−2^ to (1 × 10^7^) cm^−2^ when varies the Te concentration from 6% to 35%, and that the Fermi level for all samples with *x* ≤ 35% was below *E*_c_ at room temperature. This analysis suggests that the carrier densities in the semiconducting 1H‐WTe_2_ *_x_* S_2(1‐_ *_x_*_)_ alloys may be controlled by tuning the Te doping level.

The optical properties of the WTe_2_ *_x_* S_2(1‐_ *_x_*_)_ alloys were investigated by Raman and PL spectra, and the *E*_emission_ values derived from the PL spectra were applied to estimating the (*E*_c_ − *E*_F_) values and the electron doping concentrations using Equations (2) and (3) as stated above. In Figure 3a, Raman spectra of the WTe_2_ *_x_* S_2(1‐_ *_x_*_)_ alloys with various Te concentrations were collected to examine the composition‐dependent lattice vibrational modes. For monolayer 1H‐phase WS_2_, the two characteristic peaks $E_{2g}^{1}$and *A*_1_ *_g_* were located at 351 cm^−1^ and 419 cm^−1^, respectively, in agreement with previous reports.^[^ ^13^ ^]^ In the 1H‐phase alloys, it was evident that the Raman footprints changed with increasing Te concentration relative to those of the pure WS_2_, where the 1H‐phase characteristic peaks weakened and additional peaks associated with the 1T′‐phase appeared around 163 cm^−1^ and 213 cm^−1^. The positions of the two WS_2_ vibrational modes were softened and redshifted with the increase of Te concentration, which may be attributed to the effect of heavier Te atoms on decreasing the vibrational frequencies. In comparison with pure 1T′‐WTe_2_ with main *A*_1_ modes^[^ ^48^ ^]^ at 120, 132, 162, and 213 cm^−1^, the observed new peaks around 195, 225, 290, and 400 cm^−1^ in Figure 3a were similar to the 1H‐phase and 1T′‐phase WS_2_‐like peaks reported previously.^[^ ^49^, ^50^, ^51^, ^52^, ^53^ ^]^

In addition to the Raman spectra, PL measurements were performed on the alloys to investigate the composition‐dependent optical bandgap (*E*_emission_) evolution and phase transition in Figure 3b. We found that the optical bandgap of the WTe_2_ *_x_* S_2(1‐_ *_x_*_)_ alloys could be tuned from 2 eV (for pure 1H‐WS_2_) to zero (for pure 1T′‐WTe_2_) as the concentration of Te increased, and 1H to 1T′ phase transition existed at an intermediate Te concentration (*x* > 0.35) in the WTe_2_ *_x_* S_2(1‐_ *_x_*_)_ alloys. For 1T′ ternary tellurides, no PL signal could be detected because of their metallic nature. Notably, within the 1H phase, the correlation between the optical bandgap and the Te concentration was approximately linear to each other, and the 1H‐phase optical bandgap ranged between 2 eV (pure WS_2_, *x* = 0) and 1.75 eV (WTe_2_ *_x_* S_2(1‐_ *_x_*_)_ alloy, *x* = 0.35), as presented in the inset of Figure 3b. Additionally, the composition‐dependent PL peak position of the as‐grown alloys was found to be in good agreement with the quadratic rule of the bandgap (*E*_g_) estimation reported by Kang et al:^[^ ^54^ ^]^

(4) $$E_{g}\left({\mathrm{WTe}}_{2x}S_{2(1-x)}\right)=xE_{g}\left({\mathrm{WTe}}_{2}\right)+\left(1-x\right)E_{g}\left({\mathrm{WS}}_{2}\right)-bx\left(1-x\right)$$

where the parameter *b* for WTe_2_ *_x_* S_2(1−_ *_x_*_)_ alloy equals 0.08,^[^ ^54^ ^]^ and the bandgap of the 1H‐phase WTe_2_ is 1.03 eV from literature.^[^ ^7^, ^55^, ^56^ ^]^

The validation of the correlation between the *E*_emission_ value and the Te doping level in Equation (4) thus provides a fast and efficient way to determine the chemical composition of the WTe_2_ *_x_* S_2(1‐_ *_x_*_)_ alloy. The neutral A‐exciton PL peak of different WTe_2_ *_x_* S_2(1‐_ *_x_*_)_ alloys, which resulted from direct‐gap A‐exciton recombination at the K/K′ points in the Brillouin zone, exhibited an approximately 250 meV redshift when doped with ≈ 35% Te. We also fitted the PL spectra of pure WS_2_ and the 1H‐phase WTe_2_ *_x_* S_2(1‐_ *_x_*_)_ alloys (*x* < 0.5) to deconvolve (Supporting Information, Figure S2) the A‐exciton and trion contributions, and found that the optimal lineshape for the spectral contributions was a mixed Gaussian–Lorentzian function. As shown in Figure 3c, the A‐exciton and trion peaks of WTe_2_ *_x_* S_2(1‐_ *_x_*_)_ alloys (*x* < 0.5) were both redshifted relative to those of pure WS_2_, which was consistent with the decreasing optical bandgap with increasing Te doping.

Figure 3d–i show the polarization‐dependent PL spectra of 1H phase monolayer WS_2_ and WTe_2_ *_x_* S_2(1‐_ *_x_*_)_ alloys on SiO_2_/Si substrate under *σ* ^+^ circularly polarized excitation. The valley polarization of WS_2_ at room temperature (RT) rarely exceeded 5%, but the DVP in monolayer ternary WTe_2_ *_x_* S_2(1‐_ *_x_*_)_ (*x* < 0.5) alloys were found to vary from 3% (for *x* = 35%) to 40% (for *x* = 6%). The significant enhancement in the valley‐polarization at RT from WS_2_ to WTe_0.12_S_1.88_ may be attributed to the enhanced spin‐orbit coupling by introducing Te atoms in WS_2_ lattice. On the other hand, the substitutions of S atoms by Te atoms also markedly affect the carrier density. The estimated 2D carrier density versus Te‐concentration is shown in Figure 2j, showing an initial rapid increase from *x* = 0 to *x* = 6% followed by a monotonic decreasing trend with a further increase in the Te‐concentration. The Te‐doping dependence of the 2D carrier density is similar to that of the DVP shown in Figure 3i, where the highest enhancement in the valley polarization (≈ 40%) at RT was found when the carrier density reached the highest value (≈3 × 10^10^) cm^−2^ in the WTe_0.12_S_1.88_ (*x* = 6%) alloy. Similar behavior of the DVP dependence on Te doping for WTe_2_ *_x_* S_2(1‐_ *_x_*_)_ alloys is also found under *σ ^−^* circularly polarized excitation, as shown in Figure S3 (Supporting Information). This correlation between the carrier density and the DVP may be understood in terms of increasing exciton screening effects with increasing carrier densities, which resulted in reduced long‐range electron‐hole exchanging interactions and hence suppressed the momentum‐dependent intervalley scattering and improved the DVP.

Noting the benefits of carrier doping and increased spin‐orbit coupling on enhancing the DVP in WTe_0.12_S_1.88_, we conjectured that further enhancement of the DVP may be achieved by controlling the carrier densities via electrostatic doping, which would require the development of high‐quality electrical contacts with reduced SBH and weakened Fermi level pinning to the 1H‐WTe_2_ *_x_* S_2(1‐_ *_x_*_)_ alloys. To this end, we fabricated back‐gated FETs based on WTe_2_ *_x_* S_2(1‐_ *_x_*_)_ alloys on (P++)Si/SiO_2_ substrates and used specially designed electrical contacts to evaluate the performance of these devices, which provided critical information about the quality of our electrical contacts on the WTe_2_ *_x_* S_2(1‐_ *_x_*_)_ alloys.

**Figure 4:**
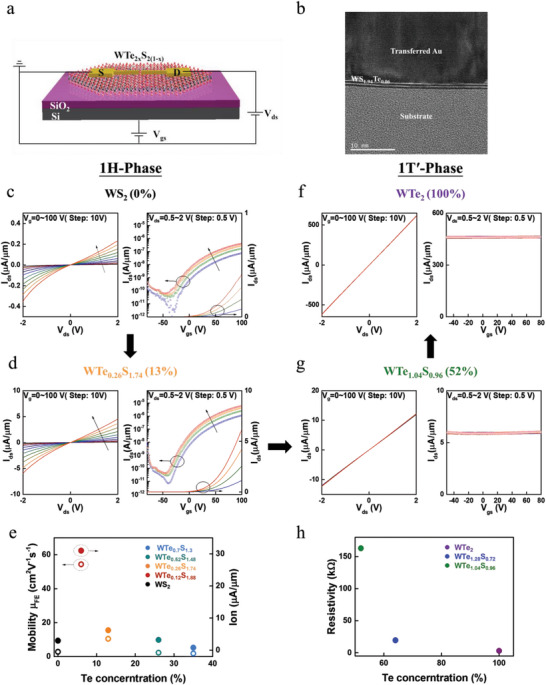
Transport characterizations of back‐gated FET devices based on 1H‐phase and 1T′‐phase WTe_2_ *_x_* S_2(1‐_ *_x_*_)_ alloys: a) Schematic illustration of a fabricated FET device. b) Cross‐sectional image of the metal‐semiconductor interface captured by transmission electron microscopy (TEM). Current‐voltage (*I*_ds_ vs. *V*_ds_) characteristics with different bottom gate voltage (*V*_g_) and gating response (*I*_ds_ vs. *V*_gs_) with different source‐drain voltages (*V*_ds_) from 0.5 V to 2 V for c) 1H‐WS_2_, and d) 1H‐WTe_0.26_S_1.74_. e) Room‐temperature µ_FE_ and *I*_on_ of monolayer semiconducting WTe_2_ *_x_* S_2(1‐_ *_x_*_)_ alloys with *x* = 0, 0.06, 0.13, 0.26, and 0.35. Current‐voltage (*I*_ds_ vs. *V*_ds_) characteristics with different bottom gate voltage (*V*_g_) and gating response (*I*_ds_ vs. *V*_gs_) with different source‐drain voltages (*V*_ds_) from 0.5 V to 2 V for f) 1T′‐WTe_1.04_S_0.96_ and g) 1T′‐WTe_2_. h) Room‐temperature resistivity of monolayer metallic WTe_2_ *_x_* S_2(1‐_ *_x_*_)_ alloys with *x* = 0.52, 0.64, and 1.

To fabricate 1H‐WTe_2_ *_x_* S_2(1‐_ *_x_*_)_ based FETs, monolayer WTe_2_ *_x_* S_2(1‐_ *_x_*_)_ flakes were first transferred to heavily p‐doped Si substrates with a SiO_2_ top layer of 285 nm thickness, which served as a bottom gate and a gate dielectric, respectively. The metallic contact electrodes were fabricated by E‐beam lithography, and 200 nm Au contact electrodes were deposited on silanol functionalized SiO_2_/Si substrate using E‐beam evaporation. The channel length (*L*) and width (*W*) of the fabricated devices were 0.5 µm and 1 µm (**Figure** **4**a), respectively. The metallic contact electrodes were transferred and aligned on top of the WTe_2x_S_2(1‐x)_ monolayer flake by means of a metal transferred method, to be elaborated in the experimental section. Using cross‐sectional analysis by transmission electron microscopy (TEM), we examined the interface between the transferred Au and the monolayer WTe_2_ *_x_* S_2(1‐_ *_x_*_)_ as shown in Figure 4b, and found that in contrast to the direct Au deposition onto monolayer TMD via electron‐beam evaporation, our process of transferring Au contacts to WTe_2_ *_x_* S_2(1‐_ *_x_*_)_ did not incur any damages to the WTe_2_ *_x_* S_2(1‐_ *_x_*_)_ layer, as evidenced by the perfect rows of atoms clearly visible in the TEM image (Figure 4b). The electrical performance of the CVD‐grown WTe_2_ *_x_* S_2(1‐_ *_x_*_)_ alloys with metal‐transferred Au contact electrodes was investigated by studying the backgated FETs made of monolayer WTe_2x_S_2(1‐x)_ alloys. The transfer characteristic curves of the monolayer WTe_2_ *_x_* S_2(1‐_ *_x_*_)_ devices are presented in Figure 4 and Figure S4, Supporting Information. In Figure 4c–e, all semiconducting 1H‐phase WTe_2_ *_x_* S_2(1‐_ *_x_*_)_ alloys (*x* = 0, 0.06, 0.13, 0.26, and 0.35) devices showed typical n‐type transport behavior with high on/off (> 10^5^) current ratios. Additionally, the field‐effect mobility (μ_FE_) may be evaluated by the following relation:^[^ ^57^, ^58^ ^]^

(5) $$\mu_{\mathrm{FE}}=\frac{L}{WV_{\mathrm{ds}}C_{g}}\frac{dI_{\mathrm{ds}}}{dV_{\mathrm{gs}}}$$

where *I*_ds_ is the source‐drain current, *V*_gs_ the gate‐source voltage; *V*_ds_ the source‐drain voltage, and *C*_g_ the gate capacitance. Using Equation (5) and the transfer characteristic curves in Figure 4c–e, we obtained mobility values of 0.58 cm^2^ V^−1^ s^−1^, 35 cm^2^ V^−1^ s^−1^, 10.5 cm^2^ V^−1^ s^−1^, 2.8 cm^2^ V^−1^ s^−1^, and 1.8 cm^2^ V^−1^ s^−1^ for the 1H‐phase WTe_2_ *_x_* S_2(1‐_ *_x_*_)_ alloys with *x* = 0, 0.06, 0.13, 0.26, and 0.35, respectively. The ON current (*I*_on_) for the WTe_0.12_S_1.88_ alloy‐based devices was improved by 2 orders of magnitude relative to the control devices (WS_2_‐FET devices). In contrast, for the 1T′‐phase alloys, the drain current was found to increase by ≈50 times in magnitude from the 1T′‐phase WTe_2_ *_x_* S_2(1‐_ *_x_*_)_ alloys (*x* = 0.52) to pure WTe_2_, which implied that the metallic behavior of tellurides could be modified by controlling the concentration of the alloying S atoms. Furthermore, the source‐drain current (*I*_ds_) was completely independent of the backgated voltage (*V*_gs_) in all three semimetallic 1T′‐phase WTe_2_ *_x_* S_2(1‐_ *_x_*_)_ alloys (*x* = 0.52, 0.64 and 1), as shown in Figure 4f–h, and the resistivity of the WTe_2_ devices was reduced by 2 orders of magnitude as compared to that of the 1T′‐phase WTe_2_ *_x_* S_2(1‐_ *_x_*_)_ (*x* = 0.52) devices.

The improvement in the electrical performance of the FETs based on the 1H‐phase semiconducting WTe_2_ *_x_* S_2(1‐_ *_x_*_)_ alloys with Au electrode can be attributed to several effects. At the metal‐semiconductor interface, electrons can be injected from the metal to the semiconductor either by thermionic emission over the Schottky barrier or via tunneling through the Schottky barrier. The width of the Schottky barrier is equal to the width of the depletion region (*W*_dep_), which depends on the doping concentration (*N*_D_) of the semiconductor and is proportional to (*N_D_*)^−1/2^according to the following relation: $W_{\mathrm{dep}}=\sqrt{2\varepsilon_{s}V_{\mathrm{bi}}d_{\mathrm{TMD}}/\left(eN_{D}\right)},$ where *d*_TMD_ ∼ 0.6 nm is the monolayer thickness of the TMD sample, *ε_s_* (≈5) is the dielectric constant of semiconductors, *V*_bi_ = (*ϕ*_M_ − *ϕ*_s_)/*e* is the built‐in potential between the metallic contact and the TMD semiconductor, and *e* is the elementary charge. Therefore, *W*_dep_ decreased with increasing *N*_D,_ and the probability of electron injection into the semiconductor via tunneling through the Schottky barrier increased. Indeed, we found the largest *I*_on_ and highest *µ*_FE_ in the lightly Te‐doped FET devices (WTe_2_ *_x_* S_2(1‐_ *_x_*_)_ with *x* = 6%) because of the maximum *N*_D_ value ∼(3 × 10^10^) cm^−2^ that induced the minimum depletion width *W*_dep_, which enhanced the electron tunneling through the Schottky barrier. Additionally, we note that the quality of the electrical contact between the metallic electrode and the semiconducting channel directly affects the carrier injection and therefore the performance of the devices. In the case of TMD‐based devices, at the metal electrode/TMD interface, the large bandgap of TMDs leads to a Schottky barrier (SB) and a van der Waal (vdW) gap without chemical bonds, which gives rise to a high contact resistance for the as‐fabricated devices. Thus, it is imperative to eliminate the interfacial vdW gap and to depin the Fermi level of the metallic electrode to facilitate efficient charge transport across the contact interface for optimized FET device performances as well as efficient control of electrostatic doping.

To overcome the Fermi level pinning effect and to lower the Schottky barrier height (SBH) at the interface, we developed a new process to transfer surface‐functionalized, water‐assisted wafer Au electrodes onto monolayer WTe_2_ *_x_* S_2(1‐_ *_x_*_)_ to form WTe_2_ *_x_* S_2(1‐_ *_x_*_)_‐based FETs with Au contacts, as schematically illustrated in Figure S5. Besides using the Au contacts, there are two known methods to date for eliminating the Fermi‐level pinning effects of electrical contacts to TMDs. One is to strengthen the hybridization by doping the underlying TMDs. The other is to weaken the hybridization between the contact electrode and the TMDs by inserting graphene to greatly reduce the contact resistance and SBH. The use of heterostructures that consist of a 2D van der Waals (vdW) semi‐metal, such as graphene, as the top contact material on a 2D semiconductor, is a common approach to lower the SBH and contact resistance. However, deposition of another metallic layer on graphene is required for electrical characterizations, and the carrier injection efficiency generally varies, depending on the metal deposited on graphene. Alternatively, the metallic 1T′‐phase WTe_2_ with a low work function and a vdW clean surface may be an efficient electron‐type (*n*‐type) contact material for 2D semiconductors. However, there have not been extensive studies to date on using the 1T′‐phase WTe_2_ as the metal contact to lower the contact resistance of TMD‐based devices because of the challenges in materials preparation and stability. Noting that Te‐based monolayers are known to be unstable in ambient conditions, we chose multilayer WTe_2_ as the electrodes alternative to Au contacts for the WTe_2_ *_x_* S_2(1‐_ *_x_*_)_ alloy‐based FET devices. The stability test of multilayer WTe_2_ is illustrated in Figure S6 (Supporting Information), which demonstrates that multilayer WTe_2_ could be stable in air beyond 15 days.

**Figure 5:**
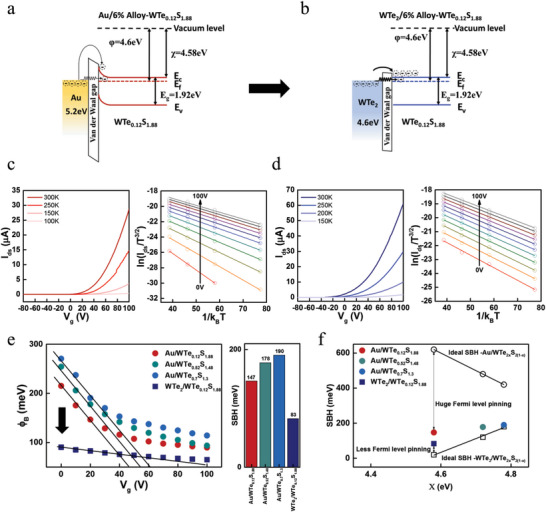
Schottky barrier height between metal electrodes (Au, WTe_2_) and WTe_2_ *_x_* S_2(1‐_ *_x_*_)_ alloys: a) Schematic band diagram of WTe_2_ *_x_* S_2(1‐_ *_x_*_)_ FET interface with Au electrode obtained from UPS measurements. b) Schematic band diagram of WTe_2_ *_x_* S_2(1‐_ *_x_*_)_ FET interface with 1T′‐WTe_2_ electrode obtained from UPS measurements. Temperature‐dependent two‐terminal current *I*_ds_ as a function of *V*_gs_ at *V*_ds_ = 2 V for the FET devices with c‐left) Au, and d‐left) WTe_2_ electrical contacts. Gate‐voltage‐dependent ln (*I*_0_/*T* ^3/2^) versus (*1*/*kT*) plot with two different contacts: c‐right) Au, and d‐right) WTe_2_. From the slopes of the curves shown in Figure c), d) and Figure S7, the gate‐voltage‐dependent SBH (*ϕ*_B_) of the FETs are extracted in e). f) Comparisons of the extracted SBH at *V*_FB_ of the monolayer 6%‐WTe_2_ *_x_* S_2(1‐_ *_x_*_)_ alloys with WTe_2_ electrode and the monolayer WTe_2_ *_x_* S_2(1‐_ *_x_*_)_ alloys with *x* = 0.06, 0.26, and 0.35 and Au electrode.

Next, we investigated the characteristics of the 1H‐WTe_2x_S_2(1‐x)_‐based FETs with two types of transferred source(S)/drain(D) electrodes: Au (work function ≈ 5.2 eV), and 1T′‐WTe_2_ (work function ≈ 4.6 eV), as shown in **Figure** **5**. Given that 1T′‐WTe_2_ has the closest electron affinity to the work function of 1H‐WTe_0.12_S_1.88_, we expected the use of 1T′‐WTe_2_ electrodes to induce the lowest Schottky barrier height. The work function of each electrode was measured by ultraviolet photoelectron spectroscopy (UPS) and the results are shown in Figure S7, Supporting Information. Figure 5a,b illustrate the band diagrams of the Au/1H‐WTe_0.12_S_1.88_ and 1T′‐WTe_2_/1H‐WTe_0.12_S_1.88_ interfaces in the equilibrium condition after both of the contacts were made. The charge injection in the 2D WTe_0.12_S_1.88_ channel was determined by the SBH and Schottky barrier width (SBW), both largely dependent on the extent of the semiconductor band‐bending at the metal (Au or 1T′ WTe_2_) and 1H‐WTe_0.12_S_1.88_ Schottky contact region. While the SBH governed the extent of thermionic emission of carriers over the barrier, the SBW determined the extent of the thermionic field emission and quantum tunneling of charge carriers. Hence, both the SBH and SBW must be minimized to achieve efficient injection of charge carriers from the contact into the semiconducting WTe_0.12_S_1.88_ channel as shown in Figure 5b.

The field‐effect mobility and the on/off current ratios of 1H‐phase WTe_0.12_S_1.88_ crystal for Au and WTe_2_ electrodes were found to be *µ*_FE_ = 35 cm^2^ V^−1^ s^−1^ and (*I*_on_/*I*_off_) = 5 × 10^5^, and *µ*_FE_ = 50 cm^2^ V^−1^ s^−1^ and (*I*_on_/*I*_off_) = 1.1 × 10^6^, respectively. In particular, we found substantially more efficient gate tunability in the FET with 1T′‐WTe_2_ contacts. That is a smaller threshold voltage *V*_g,th_ of 18 V (compared with *V*_g,th_ = 50 V for Au contacts) and a higher on‐current of ≈50 µA µm^−1^ (compared with ≈30 µA µm^−1^ for Au contacts). These findings implied that the types of electrical contacts could substantially modify the FET characteristics.

To quantitatively investigate the SBH, the FET output characteristics were measured at different temperatures (200–300 K) and presented in Figure S8. The SBH *ϕ*_B_ can be extracted from the data by using the following thermionic emission model:^[^ ^59^, ^60^, ^61^, ^62^ ^]^

(6) $$\ln\left(\frac{I_{0}}{T^{3/2}}\right)=\ln\left(AA^{*}\right)-\frac{e\phi_{B}}{k_{B}T}$$

where *A* is the junction area, A^*^ is the effective Richardson‐Boltzmann constant given by$A^{*}=4\pi em_{n}k_{B}^{2}/h^{3}$, *m*_n_ is the electronic effective mass of WTe_2x_S_2(1‐x)_, and the effective “emission current” *I*_0_ is obtained from the *I*_ds_‐versus‐*V*_ds_ curves measured at different temperatures and gate voltages. Thus, we obtained ϕ_*B*_ at the two contacts/ WTe_2_ *_x_* S_2(1‐_ *_x_*_)_ (*x* < 0.5) interfaces from the slop of the linear fit to ln (*I*_0_/*T* ^3/2^) as a function of 1/(*k*_B_ *T*) (Figure 5c,d and Figure S7, Supporting Information). In Figure 5e, the effective SBH were extracted under the flat band gate voltage (*V*_g_) condition, which corresponded to the start of deviation of the ϕ_*B*_ versus *V*_g_ curve from the linear slope. Figure 5f summarizes the relation between the SBH (at the *V*_FB_) of the metal‐semiconductor junction (MSJ) and the work functions of the Au and WTe_2_ in contact with monolayer WTe_2_ *_x_* S_2(1‐_ *_x_*_)_ alloys. The SBH between the Au electrode and WTe_0.12_S_1.88_ alloy was ≈150 meV, which confirmed the existence of Fermi‐level pinning compared to the ideal Schottky‐Mott rule theoretically calculated SBH (≈620 meV). In contrast, in the case of WTe_2_ electrodes, the value of the SBH (≈80 meV) between 1T′‐WTe_2_ and WTe_0.12_S_1.88_ alloy was much closer to the ideal Schottky‐Mott rule theoretically calculated SBH (≈20 meV) for monolayer WTe_0.12_S_1.88_‐based FETs with 1T′‐WTe_2_ contacts. This finding revealed that WTe_2_ electrical contacts weakened the Fermi level pinning and thus improved the electron charge injection to the WTe_0.12_S_1.88_ alloy substantially. Additionally, the SBW for the 1T′‐WTe_2_ contact can be estimated by using the built‐in potential *V*_bi_ = (*ϕ*_M_ − *ϕ*_s_)/*e* = 0.02 and *N*_D_ ≈ (1.3 × 10^11^) cm^−2^ for the WTe_0.12_S_1.88_ alloy, which yields a small SBW ≈2.2 nm for 1T′‐WTe_2_ on WTe_0.12_S_1.88_.

**Figure 6:**
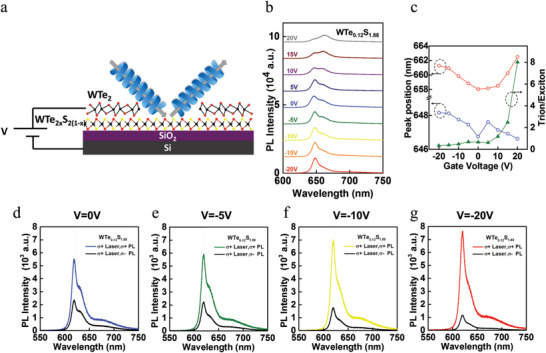
Electrically tunable valley polarization of WTe_0.12_S_1.88_ /1T′‐ WTe_2_ with lowest SBH. a) Schematic illustration of a WTe_2_/WTe_0.12_S_1.88_ heterostructure. b) PL spectra at RT for *V*_g_ values ranging from −20 V to 20 V, with an increment of 5 V. c) Gate voltage dependence of the PL peak position for neutral excitons (blue) and trions (red) under *V*_g_ from −20 V to 20 V. The intensity ratio of trions and excitons (green) is also shown as a function of *V*_g_ from −20 V to 20 V. The *σ* ^+^ (blue) and *σ* ^−^ (black) PL intensity were taken at RT under d) *V*_g_ = 0, The *σ* ^+^ (green) and *σ* ^−^ (black) PL intensity were taken at RT under e) *V*_g_ = −5 V. The *σ* ^+^ (yellow) and *σ* ^−^ (black) PL intensity taken at RT under f) *V*_g_ = −10 V. The *σ* ^+^ (red) and *σ* ^−^ (black) PL intensity taken at RT under g) *V*_g_ = −20 V.

**Figure** **6**a shows a schematic of a back‐gated FET device based on monolayer WTe_0.12_S_1.88_ alloy with WTe_2_ electrode. For a given gate voltage, there were two well‐defined PL spectral components associated with the emission bands of the neutral excitons (X) and the negatively charged trions (X^−^). We found that the emission near 650 nm (≈1.91 eV) from neutral excitons (X) was dominant around the charge neutrality point at *V*_g_ = 0. The PL spectral evolution of these two emission bands with gate voltage is illustrated in Figure 6b,c for the 1T′‐WTe_2_/1H‐WTe_0.12_S_1.88_ device. We note that the trion‐to‐exciton intensity ratios of monolayer 1H‐WTe_0.12_S_1.88_ exhibited dependence on the gate voltage, as shown in Figure 6c. The gate voltage‐dependent DVP became significantly different in the case of 1H‐WTe_0.12_S_1.88_ device with 1T′‐WTe_2_ electrodes. Figure 6b,c shows the PL spectral evolution of X and X^−^ emissions with gate voltage from the 1H‐WTe_0.12_S_1.88_ device with WTe_2_ electrodes. Additionally, polarization‐resolved PL spectra of the 1H‐WTe_0.12_S_1.88_ device with WTe_2_ electrodes under σ^+^excitations are shown in Figure 6d–g for *V*_g_ = 0, −5 V, −10 V and −20 V, respectively. For *V*_g_ = 0, which corresponded to the valley‐polarized state in pristine 1H‐WTe_0.12_S_1.88_ alloy, excitons at the K valley were more populated under σ^+^ excitations with the DVP ≈40% as expected. By increasing the electron density via decreasing the gate voltage from 0 to −5 V, −10 V and −20 V, the difference between the σ^+^ and σ^−^ components of the PL spectra became increasingly more significant, implying enhanced valley polarization of the neutral excitons. Specifically, we found that the values of DVP for *V*_g_ = 0, −5 V, −10 V, and −20 V were ≈40%, 45%, 50%, and 70%, respectively, suggesting significantly enhanced valley polarization as the applied bias moved away from the charge neutral point. Neutral excitons are the natural low‐energy excitations of a charge‐neutral semiconductor, whereas trions are only formed in the presence of excess charge. Therefore, the intensity of trion emissions is generally dependent on the amount of excess charge in the semiconductor. For this reason, trion emissions were usually not found in the PL spectra of our CVD‐grown monolayer WS_2_ samples unless a back gate voltage was applied. For the gated samples, the PL spectra typically exhibited additional emissions at 30–60 meV below the neutral excitonic line, which may be attributed to the emission from negatively charged trions (X^−^). Thus, by simply varying the applied back gate voltage, we were able to control the ratio between neutral exciton and charged trion emissions.

To gain further insights into this behavior, we performed gate‐dependent transport measurements, using a scheme where a positive bias induced hole‐doping and a negative bias introduced electron‐doping. We observed typical *n*‐type transport behavior with on/off current ratios greater than 10^6^ at room temperature, as shown in Figure S9. The doped carrier density *n* = [(εε_0_/*t*_ox_)(*V*_g_ − *V*_CNP_)/*e*] under gate voltage *V*_g_ was estimated, where *ε* = 3.9 is the dielectric constant of SiO_2_, *t*_ox_ is the thickness of SiO_2_, and *ε*_0_ is the vacuum permittivity. As shown in Figure S9, the charge neutral point (CNP) was observed to be at 20 V so that the *n*‐type carrier concentration could be estimated, which yielded 1.5 × 10^12^ cm^−2^, 1.89 × 10^12^ cm^−2^, 2.27 × 10^12^ cm^−2^, and 3.03 × 10^12^ cm^−2^ for 0 V, −5 V, −10 V, and −20 V, respectively. These values confirmed the notion that the enhancement of valley polarization by electrostatic doping in the WTe_0.12_S_1.88_ alloy may be attributed to carrier doping‐induced suppression on the inter‐valley relaxation process, because the inter‐valley relaxation process of bright excitons is dominated by the long‐range electron‐hole (e‐h) exchange interaction, and the long‐range e‐h exchange interactions may be efficiently screened by increasing the 2D carriers in the monolayer TMD with electrostatic doping. Here the screen length is determined by the inverse of the Thomas‐Fermi wave vector^[^ ^63^ ^]^ $k_{\mathrm{TF}}\left(T,E_{F}\right)=k_{\mathrm{TF}0}\left[1-\exp(-\frac{E_{F}}{k_{B}T})\right]$, where *k*_TF0_ = *g*_s_ *g*_v_ m*e ^2^/(4πεℏ^2^) is the zero temperature Thomas‐Fermi wave vector, *g*_s_ (*g*_v_) is the degeneracy for spins (valleys), m* is the effective electron or hole mass, and *ε* is the dielectric constant. The Fermi energy *E*_F_ measured from the bottom of the conduction band (to the top of the valance band) is defined by *E*_F_ = 2π*n*ℏ^2^/(*g*_s_ *g*_v_ m*), where *n* is the doped 2D electron (hole) density. Therefore, *k*_TF_ increases rapidly with increasing *n*. In the strong scattering limit, the inter‐valley scattering rate (τ_v_)^−1^ due to e‐h exchange interaction may be approximated by the relation (τ_v_)^−1^∝(*k*_TF_)^−2^. Therefore, the inter‐valley scattering rate (τ_v_)^−1^is strongly suppressed by increasing carrier doping.^[^ ^63^ ^]^ In contrast, the intra‐valley relaxation time τ_0_ is much less affected by carrier doping, as supported by the stable linewidths and integrated intensities upon doping. Noting that the valley polarization *P*_DVP_^[^ ^64^, ^65^ ^]^ is given by

(7) $$P_{\mathrm{DVP}}=\frac{P_{0}}{1+2\left(\tau_{0}/\tau_{v}\right)}$$

with *P*_0_ being the ideal valley polarization, we find that the suppression of (τ_v_)^−1^by electrostatic doping leads to enhanced *P*_DVP_, which agrees well with our experimental observations.

## 3 Conclusion

In this work, we presented new strategies to efficiently tailor the valley‐polarized PL from semiconducting monolayer 1H‐WTe_2_ *_x_* S_2(1‐_ *_x_*_)_ at RT through chemical and electrostatic doping. We synthesized different compositions of monolayer WTe_2_ *_x_* S_2(1‐_ *_x_*_)_ alloys (0 ≤ *x* ≤ 1) by alloying Te into tungsten disulfide WS_2_ with a single‐step APCVD method, and demonstrated a structural phase transition from the 1H semiconducting phase for *x* < 0.5 to the 1T′ metallic phase for *x* > 0.5. The compositions of the WTe_2_ *_x_* S_2(1‐_ *_x_*_)_ alloys were identified by using XPS and Raman spectroscopic studies. The PL spectra revealed that the optical bandgap of the WTe_2_ *_x_* S_2(1‐_ *_x_*_)_ alloy could be tuned from 2 to 1.75 eV in the 1H‐semiconducting phase and then drop to 0 in the 1T′‐metallic phase for *x* > 0.5. Additionally, studies of the FET devices based on monolayer WTe_2_ *_x_* S_2(1‐_ *_x_*_)_ alloys confirmed that the 1H‐phase alloys were *n*‐type semiconductors and the 1T′‐phase alloys were metals. We observed drastic enhancement of the DVP at RT from ∼ 5% in WS_2_ to ∼ 40% in WTe_0.12_S_1.88_ alloy, and found that the DVP values in 1H‐WTe_2_ *_x_* S_2(1‐_ *_x_*_)_ correlated with the 2D carrier densities in the alloys. These findings may be attributed to (*i*) enhanced spin‐orbit coupling due to Te‐doping, and (*ii*) enhanced screening of the electron‐hole exchanging interactions by increasing the carrier densities that resulted in reduced inter‐valley scattering of excitons. By further applying a back‐gate bias voltage (*V*_g_) to monolayer 1H‐WTe_0.12_S_1.88_‐based FETs with 1T′‐WTe_2_ electrodes that exhibited the lowest Schottky barrier height, we were able to further enhance the DVP value from ≈40% for *V*_g_ = 0 to ≈70% for *V*_g_ = −20 V, which corroborated with the notion that excess carriers provided efficient screening of the momentum‐dependent long‐range electron‐hole exchange interaction and led to reduced intervalley scattering. The methodology described in this work thus provides a promising platform to tailor the valley degree of freedom in 1H‐TMD alloys efficiently at RT, paving ways for investigating various fundamental physical properties in 2D‐TMD materials (e.g., new types of TMD‐based Wyle semimetals, spin Hall effects, opto‐valleytronic and opto‐spintronic characteristics) and for future applications of valley‐dependent optoelectronic devices in energy‐efficient information processing.

## 4 Experimental Section

### Synthesis of WTe_2x_S_2(1‐x)_

WTe_2x_S_2(1‐x)_ was grown by atmosphere‐pressure chemical vapor deposition (APCVD). Sulfur (S) and tellurium (Te) powders were placed into two quartz boats. The amount of sulfur was fixed at 10 mg, while the amount of Te was adjusted according to the weight ratio from 1 to 10. 95 mg of WO_3_ precursor mixed with 5 mg of KI was placed in a quartz boat containing the SiO_2_/Si substrates set face‐down directly above the W source precursor, and the quartz boat was then positioned upstream at 8 cm away from the Te source. A sulfur boat was placed upstream at 18 cm away from the center of the furnace and a tellurium boat was placed downstream at 5 cm away from the S source. Next, the system was pumped down to 3 × 10^−2^ torr to eliminate air and moisture. After the system reached the base pressure, the Ar/H_2_ (80/40 sccm) carrier gas was introduced until atmospheric pressure was achieved. The furnace was then heated up with a ramp rate of 35 °C min^−1^ to the growth temperatures (750 to 850 °C). The S component melted at 150 °C and the Te component melted at 450 °C were sent into the furnace at the growth temperature to grow WTe_2x_S_2(1‐x)_. The sample growth procedure proceeded for 10 minutes, after which the furnace was directly opened to room temperature to stop the reaction immediately.

### Synthesis of WS_2_

Monolayer WS_2_ was grown using APCVD as previously reported. 95 mg of WO_3_ precursor mixed with 5 mg of KI was placed in a quartz boat containing the SiO_2_/Si substrates set face‐down directly above the W source precursor, and the quartz boat was then positioned at the center of the furnace. A second boat containing 100 mg S was placed upstream at 18 cm away from the W source. Next, the system was pumped down to 3 × 10^−2^ torr to eliminate air and moisture. After the system reached the base pressure, the Ar/H_2_ (80/40 sccm) carrier gas was introduced until atmospheric pressure was achieved. The furnace was then heated up with a ramp rate of 35 °C min^−1^ to the growth temperatures (750 to 850 °C). The S component melted at 150 °C was sent into the furnace at the growth temperature to grow WS_2_. The sample growth procedure proceeded for 10 minutes, after which the furnace was directly opened to room temperature to stop the reaction immediately.

### Synthesis of WTe_2_

Multilayer WTe_2_ was grown using APCVD. 120 mg of WO_3_ precursor mixed with 15 mg of KI was placed in a quartz boat containing the SiO_2_/Si substrates set face‐down directly above the W source precursor, and the quartz boat was then positioned at the center of the furnace. A second boat containing 500 mg Te was placed upstream at 10 cm away from the W source. Next, the system was pumped down to 3 × 10^−2^ torr to eliminate air and moisture. After the system reached the base pressure, the Ar/H_2_ (80/40 sccm) carrier gas was introduced until atmospheric pressure was achieved. The furnace was then heated up with a ramp rate of 35 °C min^−1^ to the growth temperatures (775 to 800 °C). The Te component melted at 450 °C was sent into the furnace at the growth temperature to grow WTe_2_. The sample growth procedure proceeded for 25 minutes, after which the furnace was directly opened to room temperature to stop the reaction immediately.

### Metal Transfer Process

A 4‐inch SiO_2_/Si wafer with a 300 nm‐thick oxide layer was first functionalized with an OH group by the following squence: The SiO_2_/Si wafers were dipped into 80°C piranha solution (H_2_SO_4_ : H_2_O_2_ = 3:1) for 2 h to render the surface of the substrates hydrophilic, which were subsequently washed with deionized water and dried with nitrogen gas. Next, the SiO_2_/Si wafers were cleaned with O_2_ plasma (300 mTorr, 10 sccm, and 100 W) for 10 min. Finally, the wafers were soaked in 60°C H_2_O_2_ solution for 60 min to become superhydrophilic on the surface. The functionalized surface of SiO_2_/Si, as schematically shown in Figure S5, Supporting Information, was used for the growth of WTe_2_ *_x_* S_2(1‐_ *_x_*_)_ alloys and for the deposition of metal electrodes. The gold deposited on the functionalized surface of SiO_2_/Si was patterned into electrodes by conventional e‐beam lithography (Figure S5c, Supporting Information). The patterned gold electrodes on the SiO_2_/Si wafer was then spin‐coated with a polystyrene (PS) or poly(methhyl methacrylate) (PMMA) layer (Figure S5d) followed by slow immersion into deionized (DI) water for the transfer (Figure S5e). Water instantly penetrated through the PS/Au or PMMA/Au stack. The growth substrate with a higher surface energy compared to the polymer/Au stack led to easy delamination and suspension of the polymer/Au stack on the surface of water. Thus, polymer‐supported wafer‐scale Au electrodes were achieved in the form of a stack, which was subsequently transferred onto the WTe_2_ *_x_* S_2(1‐_ *_x_*_)_ alloy (Figure S5e–g, Supporting Information) for the fabrication of FETs.

### XPS and UPS Measurements

XPS and UPS studies were performed under ultra‐high vacuum (residual gas pressure 5 × 10^−9^ torr) with a Kratos AXIS Ultra DLD and a magnetic immersion lens that consisted of a spherical mirror and concentric hemispherical analyzers with a delay‐line detector (DLD). An Al Kα (1.486 KeV) monochromatic source and He 1 (21.2 eV) source were used as excitation sources for the XPS and UPS measurements, respectively. Ejected electrons were collected at a 90° angle from the horizontal.

### Raman and PL Characterizations

The Raman and PL spectra were taken with a Renishaw InVia Raman spectrometer system using a 514.3 nm laser (2.41 eV) as the excitation source. A 50× objective lens with a numerical aperture of 0.75 and a 2400 lines mm^−1^ and 1800 lines mm^−1^ grating were chosen during the measurement to achieve a better signal‐to‐noise ratio.

### STEM Characterization

The cross‐sectional sample for the TEM experiments was prepared using a dual beam‐focused ion beam (FIB)/SEM system (SMI3050SE, SEIKO). HR‐TEM and SAED were performed using a field emission transmission electron microscope (JEM‐2100F, Joel) with an acceleration voltage of 100 kV.

### Device Fabrication and Measurements

WTe_2_ *_x_* S_2(1‐_ *_x_*_)_ field‐effect transistors (FETs) were fabricated using standard electron‐beam (E‐beam) lithography techniques. First, the WTe_2_ *_x_* S_2(1‐_ *_x_*_)_ monolayer flakes were transferred on a heavily p‐doped Si substrate with 300 nm thick SiO_2_ layer, which served as a bottom gate and a gate dielectric, respectively. The metallic contact electrodes were fabricated by E‐beam lithography, and 200 nm Au contact electrodes were deposited on silanol functionalized SiO_2_/Si substrate using E‐beam evaporation. The channel length (*L*) and width (*W*) of the fabricated devices were 0.5 µm and 1 µm. The metallic contact electrodes were transferred and aligned on top of the WTe_2x_S_2(1‐x)_ monolayer flake using our metal transferred method described earlier under the paragraph of metal transfer process. To transfer WTe_2_ contacts onto the WTe_2x_S_2(1‐x)_ monolayer flakes, the synthesized WTe_2_ films were first transferred onto the silanol functionalized SiO_2_/Si substrates by the PMMA‐assisted wet‐transferred method. Next, the WTe_2_ electrodes were patterned using E‐beam lithography and oxygen plasma treatment (10 sccm O_2_ gas, 20 mtorr, and 80 W) that selectively etched away the exposed WTe_2_ regions. The WTe_2_ patterns (*L* = 0.5 µm and *L* = 1 µm) were subsequently transferred onto the WTe_2x_S_2(1‐x)_ monolayer flake on a Si/SiO_2_ substrate using the transferred method. The electrical properties of WTe_2_ *_x_* S_2(1‐x)_ FETs were studied using a Keithley 2636 sourcemeter as a DC voltage source in vacuum at 200 −300 K.

## Conflict of Interest

The authors declare no conflict of interest.

## Author Contributions

W.‐H.L. and N.‐C.Y. conceived the research ideas. W.‐H.L. and C.‐S.L. contributed equally to this work. W.‐H.L. constructed the CVD system for WS_2_, WTe_2,_ and WTe_2x_S_2(1‐_ *_x_*_)_ growth and participated in all the measurements and data analysis. W.‐H.L. and H.A.A. contributed to the XPS measurement. W.‐H.L., C.‐S.L., and G.R.R. contributed to the Raman and PL mapping measurements. W.‐H.L., C.‐S.L., and C.I.W. contributed to the FET device measurements. W.‐H.L. and N.‐C.Y. wrote the manuscript, and N.‐C.Y. supervised and coordinated the project.

## Data Availability

The data that support the findings of this study are available from the corresponding author upon reasonable request.
